# Antibiotic dispensing practices and patterns of non-prescription antibiotic sales among retail drug outlets in Ghana

**DOI:** 10.1186/s12889-026-28725-y

**Published:** 2026-07-25

**Authors:** Bismark Senyo Donkor, Kingsley Badu, Kwadwo Boampong, Vivian Etsiapa Boamah, Vincent Edinam Assor

**Affiliations:** 1 School of Public Health, Kwame Nkrumah University of Science and Technology, Kumasi, Ghana; 2 Department of Theoretical and Applied Biology, Kwame Nkrumah University of Science and Technology, Kumasi, Ghana; 3 Department of Pharmaceutics, Kwame Nkrumah University of Science and Technology, Kumasi, Ghana; 4 Fred N. Binka School of Public Health, University of Health and Allied Sciences, Hohoe, Ghana

**Keywords:** Antibiotics, Non-prescription sales, Retail outlets

## Abstract

**Background:**

This study evaluated antibiotic dispensing practices and patterns of non-prescription antibiotic sales among community retail drug outlets in Ghana, to identify which dispenser cadres, client interaction behaviours, and antibiotic types are most implicated in non-prescription dispensing, to generate evidence to support the design of targeted, evidence-based interventions at the retail level.

**Methods:**

A cross-sectional study design was used. Data were collected from 158 dispensers, entered, and analyzed using STATA between April and July 2024. Descriptive statistics (mean, SD, median frequency, and percentages) were used to analyze self-reported antibiotic dispensing practices, antibiotic types dispensed, associated diseases and conditions, and influencing factors of antibiotic dispensation without prescription. Chi-square (χ²), bivariate, and multivariate logistic regression analyses identified significant predictors of antibiotic dispensing without prescription.

**Results:**

A total of 86 (54.4%) out of 158 respondents reported dispensing without a valid prescription, with 80.2% (69) of those dispensing without a prescription doing so regularly. Ciprofloxacin and amoxicillin (65.8%) were most frequently dispensed, while genitourinary infections (74.7%) were the most common disease conditions for which treatment is given before medications are used in treatment. Pharmacists (AOR = 4.63, 95% CI: 1.20-17.83; *p* = 0.026) were significantly more likely to engage in non-prescription dispensing than other dispensers. Dispensers who requested a history of the client’s history on antibiotic usage (AOR = 0.30, 95% CI: 0.10–0.89; *p* = 0.03) were less likely to dispense antibiotics without a prescription compared to those who did not request clients’ antibiotic use history. P-values less than 0.05 (*p* < 0.05) were considered statistically significant associations.

**Conclusion:**

A high rate of non-prescription antibiotic sales at retail drug outlets was identified in this study, underscoring the urgent need for regulatory enforcement and community-targeted, evidence-based interventions to promote responsible antibiotic dispensing practices at the retail level.

**Supplementary Information:**

The online version contains supplementary material available at https://doi.org/10.1186/s12889-026-28725-y.

## Background

Ghana faces a growing burden of antibiotic misuse and antimicrobial resistance. Although most antibiotics are classified as prescription-only medicines under the regulatory oversight of the Food and Drug Authority, they continue to be widely dispensed without prescriptions at community retail outlets [1]. The scale of this problem is well-documented across multiple regions of the country through a simulated client study conducted across four metropolitan cities, which found that 88.3% of sampled community pharmacies and drug outlets dispensed antibiotics without a prescription, with rates ranging from 82.4% in Tamale to 92.5% in Kumasi [2]. Over 80% of individuals who obtained antibiotics without a prescription did so directly from a pharmacy, underscoring the central role of retail outlets as the primary point of unsupervised access to antibiotics in Ghana [3].

Self-medication further compounds this burden, with high prevalence documented in urban informal settlements and reports of patients using leftover antibiotics or obtaining medicines from unlicensed sellers in Ghana [4]. Studies have reported high resistance to commonly used antibiotics, including tetracycline (82%), ampicillin (76%), chloramphenicol (75%), and cotrimoxazole (73%), with penicillin gradually losing effectiveness against Streptococcus pneumoniae and Neisseria meningitidis [5]. Common pathogens including streptococci, Salmonella, and E. coli have exhibited multiple drug resistance at rates as high as 78.7% across teaching, regional, and district hospitals in Ghana [5]. Reflecting this cumulative burden, the World Health Organization (WHO) Ghana 2023 Annual Report estimated that antimicrobial resistance was linked to 25,300 deaths in the country [6], while suboptimal antibiotic prescribing practices have been estimated at a pooled rate of approximately 45% nationally [4].

In developing countries, antimicrobial resistance is largely accelerated by inappropriate self-medication [6–9].

Retail drug outlets, which are community-based medicine-dispensing establishments, including licensed community pharmacies, licensed chemical shops, and patent medicine vendors, represent the most accessible point of antibiotic access for most populations in low- and middle-income countries, including Ghana [10]. The personnel working within these outlets, including pharmacists, medicine counter assistants, pharmacy assistants and technicians, and shop attendants, collectively referred to as drug dispensers, are responsible for the day-to-day dispensing of medicines to the public [11]. While these providers play a vital role in expanding healthcare access, they are also recognized as significant contributors to antimicrobial resistance through inappropriate antibiotic dispensing without valid prescriptions [7, 12, 13]. In Ghana, the retail pharmaceutical sector operates across three tiers, including licensed community pharmacies, licensed chemical shops, and an informal medicine retail sector, each functioning under differing levels of prescription enforcement [14]. This structural variability, combined with high demand for self-medication, has contributed to Ghana recording one of the highest rates of antibiotic consumption in sub-Saharan Africa [1, 6].

Studies conducted in Ghana have documented widespread dispensing of antibiotics without prescription across different regions, with non-prescription antibiotic sales reported at rates exceeding 50% in some settings [14–16]. These dispensing patterns are compounded by a fragmented regulatory system in which licensed pharmacies, licensed chemical shops, and unlicensed retail outlets operate under varying levels of oversight, creating inconsistent adherence to prescription requirements across dispenser cadres in Ghana [17].

Despite growing evidence of the problem, critical gaps remain in understanding the specific dispensing behaviours, dispenser-level factors, and client interaction practices that drive non-prescription antibiotic sales in Ghana’s retail drug outlet sector. A few studies have examined self-medication and antibiotic use behaviourally, but have not systematically addressed dispensing patterns or the factors that influence dispensing decisions at the retail level [14–16]. Addressing this gap is essential to move beyond describing the problem toward designing interventions that target the specific behaviours and structural factors involved [17–19]. This study therefore assessed antibiotic dispensing practices and patterns of non-prescription antibiotic sales among retail drug outlets in Ghana, and identified dispenser-level predictors of non-prescription dispensing, to generate evidence to support the design of targeted, context-specific interventions at the retail level in Ghana.

## Methods

### Study site

This study was conducted in the Greater Kumasi Metropolis, the administrative capital of Ghana’s Ashanti Region. The metropolis spans approximately 213 km² and has a population exceeding 2.6 million. Administratively, it comprises seven divisions: Kumasi Metropolitan Assembly (including Bantama, Subin, Manhyia North and South, and Nhyiaeso), Asokore Mampong, Asokwa, Suame, Kwadaso, Tafo, and Oforikrom municipalities [20].

The study site was selected due to its high concentration of retail drug outlets and its role as a major urban and commercial hub, which makes it a suitable setting for examining antibiotic dispensing practices. The diversity of healthcare providers and patient populations within the metropolis enhances the representativeness of findings for similar urban settings in Ghana. Additionally, the area allowed for adequate coverage of different types of drug outlets.

### Study population

The study population consisted of licensed retail community drug outlets (including pharmacies, over-the-counter medicine sellers, and herbal shops) located within the selected areas of the Greater Kumasi Metropolis. The unit of analysis was the drug dispenser present at each outlet during data collection. Drug dispensers are eligible participants, including pharmacists, pharmacy assistants and technicians, medicine counter assistants, and other dispensing staff involved in the sale of medicines. Information on provider characteristics collected included age, sex, professional qualification, years of practice, and type of retail facility. Only drug dispensers who were present at the time of data collection and provided informed consent were included. Outlets without available drug dispensers were excluded.

### Study design

A cross-sectional design was used to provide a snapshot of antibiotic dispensing practices and associated factors among drug dispensers at a single point in time. This design allowed to estimate the prevalence of dispensing without prescription and examine relationships between dispenser characteristics and behaviors. It is efficient, cost-effective, and suitable for studies conducted across multiple outlets within a limited time frame. It also fits the study objective, which focuses on describing current practices.

### Sampling

In the first stage, five of the ten sub-metropolitan areas were randomly selected as clusters. In the second stage, a sampling frame comprising all 230 registered retail drug outlets within the selected clusters was obtained from the Pharmacy Council of Ghana. The required number of outlets was allocated proportionally to each cluster based on the total number of eligible outlets. Selected outlets were then chosen using simple random sampling through the lottery method. Within each selected outlet, one eligible drug dispenser was recruited for participation.

### Sample size determination

The sample size was calculated using Cochran’s formula for finite populations [21]. An initial sample size of 385 was assumed for large populations [21]. This was adjusted using a finite population correction based on the number of registered retail drug outlets (*N* = 230), yielding a minimum sample size of 144. To account for a potential 10% non-response rate, the sample size was increased to 158 outlets. Thus, a total of 158 retail drug outlets were targeted for inclusion in the study.

### Data collection

Data were collected between April and July 2024 through interviewer-administered structured questionnaires. Interviews were conducted with drug dispensers in their respective retail drug outlets by trained research assistants under the supervision of the principal investigator. Each interview lasted approximately 15–20 min. The questionnaire was developed based on relevant literature and study objectives and was pretested among a small group of drug dispensers outside the study area to ensure clarity, relevance, and reliability. Minor revisions were made following the pretest. Participants were assured of confidentiality and anonymity, and no identifying information was recorded. The questionnaire was administered in English, with translation into the local dialect provided where necessary to enhance understanding. The questionnaire included a section allowing for multiple responses for the most common antibiotics dispensed without a prescription. Completed questionnaires were reviewed daily by the principal investigator for completeness and consistency.

### Data analysis

Data were entered into Microsoft Excel and subsequently imported into Stata version 16 for analysis. Data cleaning involved checking for missing values, outliers, and inconsistencies, which were resolved by cross-referencing with the original questionnaires. Descriptive statistics (means, medians, frequencies, and percentages) were used to summarize demographic characteristics and key study variables. Chi-square tests were conducted to assess associations between antibiotic dispensing without prescription and client behaviours, as well as other influencing factors. Bivariate logistic regression analysis was performed to identify predictors of dispensing antibiotics without a prescription. Variables that showed statistical significance were included in a multivariate logistic regression analysis. Odds ratio was used as measure of strength of association (with the accompanying p-values and confidence intervals). A p-value less than 0.05 was used as statistical significance.

### Statistical analysis

Data were analyzed using descriptive and inferential statistics. Results were summarized using frequencies and percentages for categorical variables. Associations between independent variables and the outcome were assessed using chi-square tests. Logistic regression models were fitted to identify factors associated with dispensing antibiotics without a prescription. Crude odds ratios and adjusted odds ratios were reported with 95% confidence intervals and p-values. The selection of reference categories in regression analysis was based on conceptual considerations, and consistency with established literature. Categories representing standard or baseline practice, such as Medicine Counter Assistants for job role and guideline-aligned practices for behavioral variables, were used as reference groups. This approach ensured meaningful interpretation of odds ratios and comparability with previous studies on antibiotic dispensing practices.

## Results

### Sociodemographic characteristics

Most respondents were between the ages 26 to 40 years 61.4% (*n* = 97), followed by those aged below 26 years 24.1% (*n* = 38) and those above 40 years 14.6% (*n* = 23). The mean age was 32.4 years (SD = 10.1). More than half of respondents were female 54.4% (*n* = 86), while 45.6% (*n* = 72) were male. Most respondents had tertiary education 70.9% (*n* = 112), followed by basic and high school education 20.3% (*n* = 32) and vocational education 8.8% (*n* = 14). Regarding credentials, 36.7% (*n* = 58) held Medicine Counter Assistant certificates, 30.4% (*n* = 48) held a Higher National Diploma, and 32.9% (*n* = 52) had a first degree or higher qualification. Most respondents worked in pharmacy stores 79.7% (*n* = 126), followed by over-the-counter chemical stores 17.1% (*n* = 27) and herbal shops 3.2% (*n* = 5). In terms of job roles, 41.1% (*n* = 65) were Medicine Counter Assistants, 20.3% (*n* = 32) were pharmacists and 38.6% (*n* = 61) reported other roles. Most respondents had five years or less of dispensing experience, 63.3% (*n* = 100), while 36.7% (*n* = 58) had more than five years of experience. More than half had never worked in a health facility, 55.1% (*n* = 87), while 44.9% (*n* = 71) reported prior experience in a health facility (Table 1).

**Table 1:** Sociodemographic characteristics of participants

Characteristic	Frequency (*N* = 158)	Percentage (%)
Age		
< 26 years	38	24.1
26 to 40 years	97	61.4
> 40 years	23	14.6
Mean age ± SD	32.4 ± 10.1	
Gender		
Female	86	54.4
Male	72	45.6
Education		
Basic and high school	32	20.3
Tertiary	112	70.9
Vocational	14	8.8
Credential attained for practice		
Medicine Counter Assistant Certificates	58	36.7
Higher National Diploma	48	30.4
First-degree or higher	52	32.9
Type of retail facility		
Herbal Shop	5	3.2
Over the Counter Chemical Store	27	17.1
Pharmacy Store	126	79.7
Job role		
Medicine Counter Assistant	65	41.1
Pharmacist	32	20.3
Other Staff	61	38.6
Years of dispensing experience		
≤ 5 years	100	63.3
> 5 years	58	36.7
Ever worked in a health facility		
No	87	55.1
Yes	71	44.9

### Prevalence of non-prescription antibiotic dispensing among participants

Out of the total respondents, 54.4% (*n* = 86) reported dispensing antibiotics without a prescription, while 45.6% (*n* = 72) reported not engaging in this practice. Among those who reported dispensing antibiotics without a prescription, most indicated that this occurred regularly, 80.2% (*n* = 69), while 19.8% (*n* = 17) reported doing so only occasionally (Table 2).

**Table 2:** Prevalence of non-prescription antibiotic dispensing

Variable	*n*	%
Dispensing antibiotics without prescription		
No	72	45.6
Yes	86	54.4
Frequency of non-prescription dispensing among those who dispensed without prescription		
Regularly	69	80.2
Occasionally	17	19.8

### Antibiotic dispensing practices by prescription dispensing status

Overall, 96.8% (*n* = 153) of respondents reported counselling clients on appropriate antibiotic use, including 97.2% (*n* = 70) among those dispensing with a prescription and 96.5% (*n* = 83) among those dispensing without a prescription. A total of 82.3% (*n* = 130) reported routinely requesting clients’ antibiotic use history, including 87.5% (*n* = 63) among those dispensing with a prescription and 77.9% (*n* = 67) among those dispensing without a prescription. Most respondents emphasized adherence to dosage instructions 84.8% (*n* = 134), with similar proportions among those dispensing with a prescription 84.7% (*n* = 61) and without a prescription 84.9% (*n* = 73). Only 15.2% (*n* = 24) advised clients to complete the full course of antibiotics. Regarding sources consulted before dispensing, professional consultation or client history was most common 50.0% (*n* = 79), followed by pharmacy protocols 29.1% (*n* = 46) and clinical guidelines 20.9% (*n* = 33). Use of pharmacy protocols was higher among those dispensing without a prescription 34.9% (*n* = 30), compared with those dispensing with a prescription 22.2% (*n* = 16) (Table 3).

**Table 3:** Antibiotic dispensing practices by prescription dispensing status

Variable	Total (*N* = 158) n (%)	Dispensed with Prescription (*n* = 72) n (%)	Dispensed without Prescription (*n* = 86) n (%)
Counselling clients on appropriate antibiotic use			
Yes	153 (96.8)	70 (97.2)	83 (96.5)
No	5 (3.2)	2 (2.8)	3 (3.5)
Requesting antibiotic use history from client			
Yes	130 (82.3)	63 (87.5)	67 (77.9)
No	28 (17.7)	9 (12.5)	19 (22.1)
Clients feedback on antibiotic use			
Do not complete full course	24 (15.2)	11 (15.3)	13 (15.1)
Complete Full course	134 (84.8)	61 (84.7)	73 (84.9)
Pharmaceutical references consulted prior to dispensing			
Clinical guidelines	33 (20.9)	17 (23.6)	16 (18.6)
Professional consultation or client history	79 (50.0)	39 (54.2)	40 (46.5)
Pharmacy protocols	46 (29.1)	16 (22.2)	30 (34.9)

### Dispensing behaviour by prescription dispensing status

When an antibiotic was not effective, most respondents reported substituting with an alternative 86.7% (*n* = 137), including 90.3% (*n* = 65) among those dispensing with a prescription and 83.7% (*n* = 72) among those dispensing without a prescription. A smaller proportion reported dispensing regardless 13.3% (*n* = 21), including 9.7% (*n* = 7) with a prescription and 16.3% (*n* = 14) without a prescription. When clients could not afford the prescribed antibiotic, most respondents replaced it with a cheaper alternative 80.4% (*n* = 127), including 75.0% (*n* = 54) among those dispensing with a prescription and 84.9% (*n* = 73) among those dispensing without a prescription. Others referred clients to another drug outlet 17.7% (*n* = 28), while few consulted the physician for an alternative 1.9% (*n* = 3). For handling clients requesting inappropriate antibiotics, 40.0% (*n* = 60) reported referring clients to a physician, including 44.4% (*n* = 32) among those dispensing with a prescription and 32.6% (*n* = 28) among those dispensing without a prescription. Dispensing based on history or insistence was reported by 42.4% (*n* = 67), including 38.9% (*n* = 28) with a prescription and 45.4% (*n* = 39) without a prescription. Dispensing based on knowledge or experience was reported by 19.6% (*n* = 31), including 16.7% (*n* = 12) with a prescription and 22.1% (*n* = 19) without a prescription (Table 4).

**Table 4:** Dispensing behaviour by prescription dispensing status

Variable	Total (*N* = 158) n (%)	Dispensed with Prescription (*n* = 72) n (%)	Dispensed without Prescription (*n* = 86) n (%)
Action when antibiotic will not be effective for client’s condition			
No (dispenses regardless)	21 (13.3)	7 (9.7)	14 (16.3)
Yes (substitutes)	137 (86.7)	65 (90.3)	72 (83.7)
Action when client cannot afford prescribed antibiotic			
Consult physician for an alternative	3 (1.9)	2 (2.8)	1 (1.2)
Refer to another drug outlet	28 (17.7)	16 (22.2)	12 (14.0)
Replace with cheaper alternative	127 (80.4)	54 (75.0)	73 (84.9)
Handling clients insisting on inappropriate antibiotics			
Refer to a physician	60 (40.0)	32 (44.4)	28 (32.6)
Dispense based on knowledge or experience	31 (19.6)	12 (16.7)	19 (22.1)
Dispense based on insistence	67 (42.4)	28 (38.9)	39 (45.4)

### Distribution of commonly dispensed antibiotics without a prescription

**Fig. 1:**
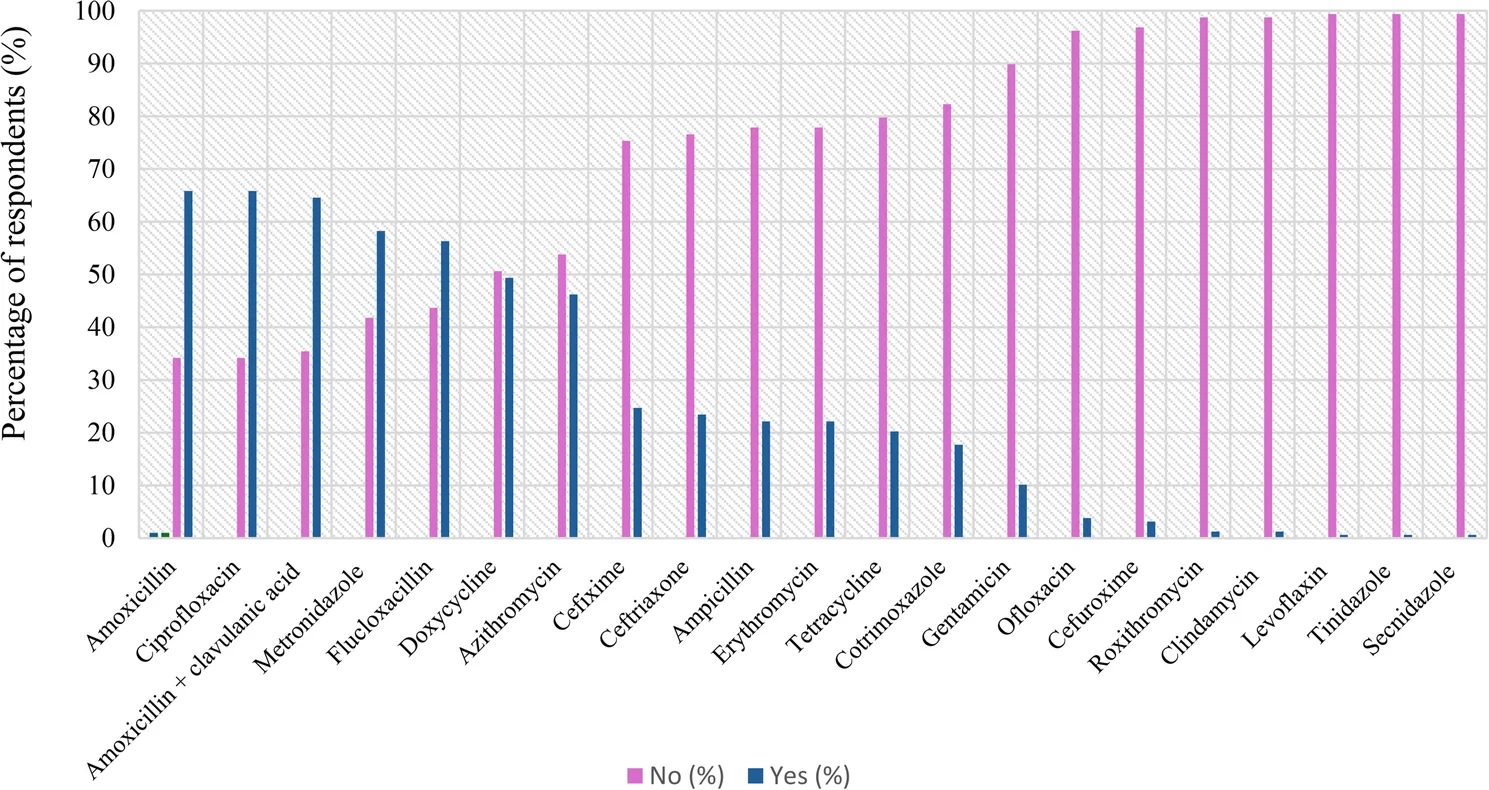
Commonly dispensed antibiotics without a prescription

Respondents were provided with a list of selected antibiotics and asked to indicate which ones they dispense without a prescription, selecting them from most frequently dispensed to least frequently dispensed. As shown in Fig. 1, ciprofloxacin and amoxicillin were the most dispensed, each reported by 65.82% (*n* = 104/158), followed closely by amoxicillin-clavulanic acid 64.56% (*n* = 102/158) and metronidazole 58.23% (*n* = 92/158). Other frequently dispensed agents included flucloxacillin 56.33% (*n* = 89/158), doxycycline 49.37% (*n* = 78/158), and azithromycin 46.20% (*n* = 73/158). Moderate levels of dispensing were recorded for cefixime 24.68% (*n* = 39/158), ceftriaxone 23.42% (*n* = 37/158), erythromycin 22.15% (*n* = 35/158), ampicillin 22.15% (*n* = 35/158), tetracycline 20.25%(*n* = 32/158), and cotrimoxazole 17.72% (*n* = 28/158). Less commonly dispensed antibiotics included gentamicin 10.13% (*n* = 16/158), ofloxacin 3.80% (*n* = 6/158), cefuroxime 3.16% (*n* = 5/158), clindamycin 1.27% (*n* = 2/158), roxithromycin 1.27% (*n* = 2/158), and several others at 0.63% (*n* = 1/158) (Fig. 1).

### Distribution of disease conditions for antibiotic dispensing without a prescription

**Fig. 2:**
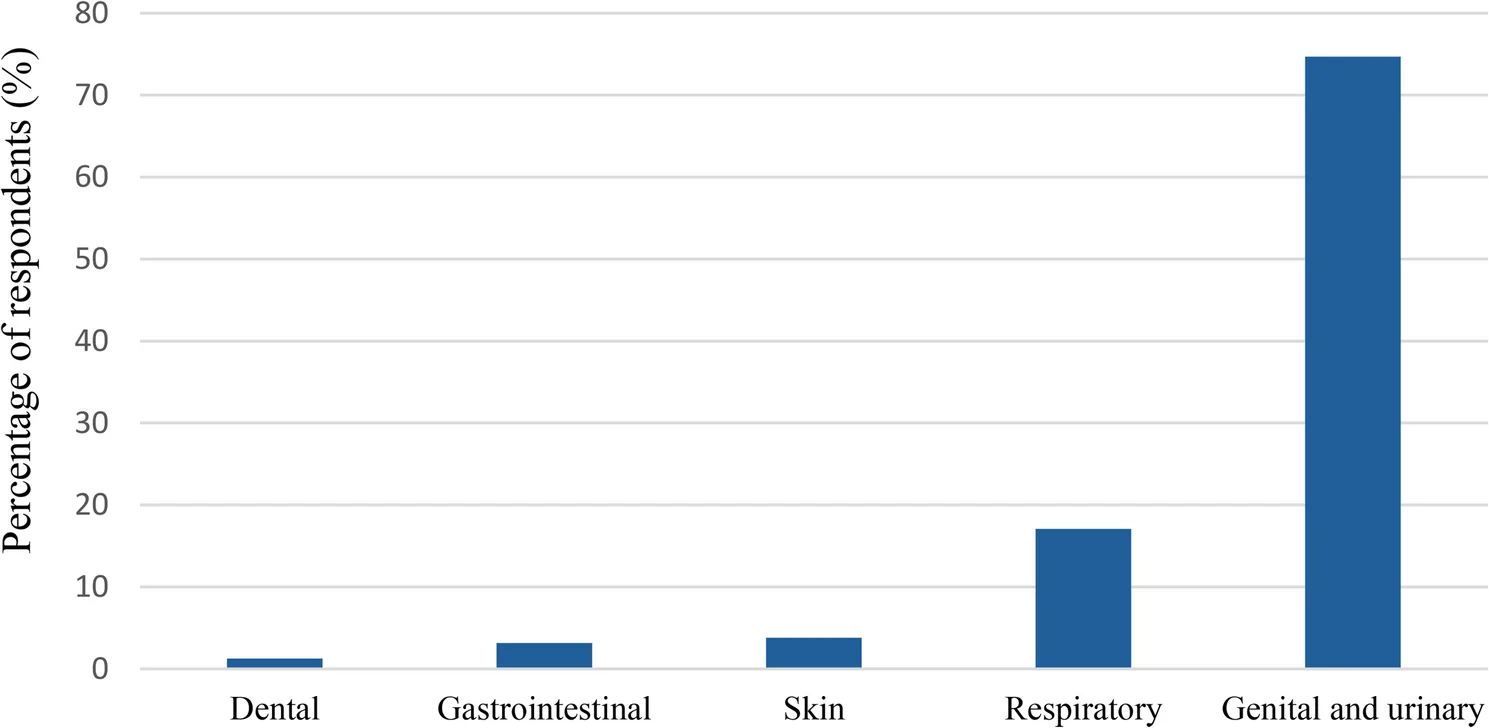
Disease conditions for dispensing antibiotics without a prescription

Figure 2 below shows the conditions under which antibiotics were most often dispensed without a prescription. Genital and urinary tract conditions dominated antibiotic dispensing, accounting for 74.7% (*n* = 118/158) of all reported cases, followed by respiratory infections, 17.1% (*n* = 27/158). Antibiotics were rarely dispensed for skin infections 3.8% (*n* = 6/158), gastrointestinal conditions 3.2% (*n* = 5/158), or dental issues 1.3% (*n* = 2/158) (Fig. 2).

### Factors influencing dispensers to dispense antibiotics without a prescription

Overall, 44.9% (*n* = 71) of respondents reported being convinced to dispense antibiotics without a prescription based on a client’s previous antibiotic use, while 32.9% (*n* = 52) dispensed antibiotics due to the perceived urgency or convenience for the client. Only 3.2% (*n* = 5) indicated that they were never convinced by clients to dispense without a prescription. Overall, 67.7% (*n* = 107) of respondents reported that they do not feel pressured by clients to dispense antibiotics. Most, 79.1% (*n* = 126), dispensed antibiotics without a prescription based on the clients’ symptoms, whereas 7.0% (*n* = 11) did so based on the clients’ ability to pay (Table 5).

**Table 5:** Factors influencing dispensers to dispense antibiotics without a prescription

Variable	Frequency (*N* = 158)	Percentage (%)
How clients convince dispensers to dispense antibiotics without a prescription		
Belief in antibiotic efficacy	30	19.0
Do not rely on clients’ complaints	5	3.2
Previous use	71	44.9
Urgency and convenience	52	32.9
Economic influence to dispense antibiotics without a prescription		
Fear of negative clients’ feedback	21	13.3
Clients’ ability to pay	11	7.0
Client’s symptoms	126	79.1
Pressure to dispense antibiotics		
No	107	67.7
Yes	51	32.3

### Determinants of antimicrobial stewardship practices in the dispensing of antibiotics

In the bivariate analysis, age and gender showed no significant association with dispensing antibiotics without a prescription (*p* > 0.05). Job role was significantly associated (χ² = 8.72, *p* = 0.013). Pharmacists had higher odds of dispensing antibiotics without a prescription compared with Medicine Counter Assistants (COR = 3.91, 95% CI: 1.49–10.32; *p* = 0.006), and this association remained significant in the adjusted model (AOR = 4.63, 95% CI: 1.20-17.83; *p* = 0.026). How respondents handled clients requesting inappropriate antibiotics was also significantly associated with the outcome (χ² = 11.52, *p* = 0.003). Compared with referring clients to a physician, dispensing based on insistence showed higher odds of dispensing without a prescription (AOR = 3.05, 95% CI: 1.32–7.04; *p* = 0.009). Dispensing based on knowledge showed elevated odds in the adjusted model but did not reach statistical significance (AOR = 2.91, 95% CI: 0.97–8.71; *p* = 0.057). Respondents who relied on client antibiotic use history showed lower odds of dispensing without prescription compared with those who did not report such practice (AOR = 0.30, 95% CI: 0.10–0.89; *p* = 0.030). Sources consulted before dispensing were also significantly associated with dispensing behavior (χ² = 12.06, *p* = 0.002). Compared with clinical guidelines, reliance on pharmacy protocols was associated with lower odds of dispensing antibiotics without prescription (COR = 0.23, 95% CI: 0.08–0.60; *p* = 0.003; AOR = 0.28, 95% CI: 0.09–0.84; *p* = 0.023). Counseling clients and pressure to dispense antibiotics were not significantly associated with dispensing behavior (*p* > 0.05) (Table 6).

**Table 6:** Determinants of antimicrobial stewardship practices in the dispensing of antibiotics

Variable	Dispensing antibiotics without a prescription		χ² (*p* value)	COR (95% CI), *p* value	AOR (95% CI), *p* value
	No *n* (%)	Yes *n* (%)			
Age			1.01 (0.605)		
Less than 26 years	20 (52.6)	18 (47.4)		Ref	Ref
26 to 40 years	42 (43.3)	55 (56.7)		1.46 (0.69–3.10), 0.329	1.40 (0.65–3.05), 0.382
More than 40 years	10 (43.5)	13 (56.5)		1.44 (0.51–4.10), 0.489	1.38 (0.47–4.05), 0.551
Gender			0.81 (0.367)		
Female	42 (48.8)	44 (51.2)		Ref	Ref
Male	30 (41.7)	39 (54.3)		1.34 (0.71–2.51), 0.368	1.29 (0.66–2.49), 0.452
Job role			8.72 (0.013)		
Medicine Counter Assistant	34 (52.3)	31 (47.7)		Ref	Ref
Pharmacist	7 (21.9)	25 (78.1)		3.91 (1.49–10.32), 0.006	4.63 (1.20–17.83), 0.026
Other staff	31 (49.2)	32 (50.8)		1.13 (0.56–2.28), 0.742	1.29 (0.56–2.96), 0.547
Counseling on antibiotic use			2.47 (0.116)		
No	4 (80.0)	1 (20.0)		Ref	Ref
Yes	68 (44.4)	85 (55.6)		5.00 (0.55–45.78), 0.154	4.12 (0.44–38.90), 0.217
Handling clients requesting inappropriate antibiotics			11.52 (0.003)		
Consult physician for an alternative	38 (63.3)	22 (36.7)		Ref	Ref
Dispense based on knowledge or experience	10 (37.0)	21 (63.0)		3.63 (1.33–9.92), 0.012	2.91 (0.97–8.71), 0.057
Dispense based on insistence	24 (35.3)	43 (64.7)		3.08 (1.48–6.41), 0.003	3.05 (1.32–7.04), 0.009
History of antibiotic use			7.34 (0.007)		
No	6 (22.2)	21 (77.8)		Ref	Ref
Yes	66 (50.7)	64 (49.3)		0.28 (0.10–0.73), 0.010	0.30 (0.10–0.89), 0.030
Pressure to dispense			0.59 (0.444)		
No	51 (47.6)	56 (52.4)		Ref	Ref
Yes	21 (41.2)	30 (58.8)		1.30 (0.66–2.55), 0.444	1.22 (0.60–2.48), 0.582
Source consulted before dispensing			12.06 (0.002)		
Clinical guidelines	10 (30.3)	23 (69.7)		Ref	Ref
Professional consultation or client history	32 (41.0)	46 (59.0)		0.62 (0.25–1.54), 0.302	0.71 (0.27–1.89), 0.494
Pharmacy protocols	30 (65.2)	16 (34.8)		0.23 (0.08–0.60), 0.003	0.28 (0.09–0.84), 0.023

## Discussion

This study highlights widespread non-prescription antibiotic dispensing and identifies the factors influencing this practice in Ghana. Most dispensers reported providing antibiotics without a prescription, a practice consistent with evidence from Ghana and other low- and middle-income countries where non-prescription antibiotic dispensing remains common despite regulatory restrictions [22–24]. Within the Ghanaian health system, this behaviour reflects weak regulatory enforcement and a central role of community pharmacies and licensed chemical sellers as accessible sources of primary healthcare. A simulated client study conducted in Ghana has consistently demonstrated that non-prescription antibiotic sales are routine across medicine retail outlets [25]. Afari-Asiedu et al. reported that clients frequently seek antibiotics from these outlets without prescriptions because they are more accessible and provide faster services than formal healthcare facilities [26]. This aligns with the present findings, where urgency and convenience were key drivers of client behaviour. Financial barriers, including consultation fees, transport costs, and time away from work, further push clients toward these outlets [15]. These structural constraints mean that seeking care at a pharmacy is often the most practical option for many patients.

The antibiotics most frequently dispensed without a prescription were ciprofloxacin, amoxicillin, amoxicillin clavulanic acid, and flucloxacillin, whereas doxycycline and azithromycin were dispensed less frequently, and antibiotics such as levofloxacin, tinidazole, secnidazole, and cefuroxime were rarely supplied. In Ghana, this pattern reflects how care is accessed and delivered through community pharmacies and licensed chemical sellers, which often serve as the first point of care [14]. Studies in Ghana have consistently reported high levels of non-prescription antibiotic sales, with commonly used antibiotics including amoxicillin and ciprofloxacin, driven by ease of access and client demand [25, 26]. The prominence of these antibiotics in the present study, therefore, reflects their availability and broad-spectrum activity in symptom-based management in settings where diagnostic capacity is limited and formal healthcare access is constrained. These patterns also align with results from Eastern Congo, where amoxicillin and ciprofloxacin were similarly identified as commonly dispensed antibiotics without prescription [22]. Dharanindra et al. reported amoxicillin as a frequently dispensed antibiotic and azithromycin as highly dispensed, in contrast to its moderate use in the present study, while ciprofloxacin and amoxicillin-clavulanic acid were among the least dispensed in their setting [27].

The high proportion of antibiotics dispensed for genitourinary infections highlights the influence of social and structural factors on treatment seeking. Genitourinary symptoms are often associated with stigma and privacy concerns, which drive clients to seek care from community pharmacies and licensed chemical sellers rather than formal health facilities. Studies in Ghana have shown that clients frequently prefer retail drug outlets for sensitive conditions due to confidentiality, shorter waiting times, and ease of access [26]. The high level of antibiotic use for these conditions, therefore, reflects both clinical need and the role of medicines outlets as discreet points of care [24, 28, 29]. Respiratory infections accounted for a substantial share of antibiotic use, which aligns with evidence from Ghana indicating frequent inappropriate antibiotic use for upper respiratory tract infections in community settings, often driven by symptom-based management and client expectations [30, 31]. The lower frequency of antibiotic use for gastrointestinal and dental conditions may reflect either reduced demand or greater reliance on alternative treatments for these conditions within the study area. Additional factors influencing this behaviour include shorter waiting times, perceived expertise of dispensers, and more approachable interactions compared to hospital settings [15].

The findings illustrate the expanding clinical role of dispensers within community medicine outlets. While limited knowledge of antimicrobial resistance and easy over-the-counter access to antibiotics have been cited in other settings [25, 27], the Ghanaian evidence suggests that access constraints, economic realities, and health system inefficiencies are more central drivers of non-prescription antibiotic use. In the present study, most dispensers reported that they would recommend an alternative antibiotic if they believed the one requested by the patient would be ineffective, and many would substitute the intended medication with a cheaper option when cost was a barrier. Similar patterns have been documented in Nepal through a study that evaluated antibiotic dispensing practices, which found that staff frequently substituted antibiotics when the preferred brand was unavailable and often directed clients to other pharmacies when necessary [28]. Comparable behaviours have been observed in Zambia, where Kalungia et al. noted that pharmacy staff commonly recommended or replaced antibiotics based on perceived effectiveness or product availability [24]. Their findings suggested that such practices may be partly driven by financial motivations rather than optimal clinical decision-making. Evidence from Saudi Arabia further supports this concern, showing that profit incentives contribute to the dispensing of broad-spectrum antibiotics not specifically requested by clients [29].

A significant association was observed between dispensing antibiotics without a prescription and the professional role of dispensers, with pharmacists being more likely to dispense without a prescription compared to other staff. This reflects professional autonomy and the expectations placed on pharmacists as accessible healthcare providers [31]. Pharmacists in community settings often serve as first contact clinicians, especially where access to physicians is limited. Their training and confidence in managing common conditions may lead to greater willingness to make independent treatment decisions, particularly in response to client demand. The evidence from this present study suggests that pharmacists frequently go beyond dispensing roles to assess symptoms, recommend therapies, and provide follow-up advice, especially in community pharmacies and licensed chemical seller outlets [30]. This expanded role is reinforced by high patient loads, long waiting times, and financial barriers in formal facilities. As a result, dispensing without a prescription can reflect responsiveness to patient needs rather than disregard for guidelines [32].

The study has been limited in a way such that a relatively small number of clusters may have reduced statistical precision due to intra-cluster correlation potentially limiting the power to detect some associations. Second, the cross-sectional design prevents causal inference between dispenser characteristics and dispensing practices. Third, dispensing practices were self-reported and may therefore be subject to recall and social desirability bias, with participants potentially underreporting behaviours that conflict with national regulations. Finally, the study did not examine in depth behaviours related to dispensing antibiotics without a prescription. Future exploratory research should assess dispensers’ views, attitudes, and knowledge on antimicrobial resistance, with a focus on their experiences, beliefs, and challenges.

## Conclusion

This study identified a high rate of non-prescription antibiotic dispensing among retail drug outlets in Ghana, with over half of dispensers reporting such practices and the majority doing so regularly. Ciprofloxacin and amoxicillin were the most frequently dispensed antibiotics, predominantly for genitourinary conditions, while pharmacists and dispensers who did not inquire about clients’ prior antibiotic use were significantly associated with non-prescription dispensing. The above findings identify specific, actionable targets for intervention, such as dispenser cadre-specific training to address non-prescription dispensing among pharmacists, reinforcement of client antibiotic history-taking as a protective dispensing behaviour, condition and antibiotic-specific regulatory attention focused on genitourinary indications. The findings provide an empirical basis to support the design of evidence-informed interventions to promote responsible antibiotic dispensing practices at the retail level in Ghana.

## Supplementary Information

Supplementary Material 1: Questionnaire.

## Data Availability

Data are available from the corresponding author upon reasonable request.
