# Supplementary material for: Antibiotic dispensing practices and patterns of non-prescription antibiotic sales among retail drug outlets in Ghana
Source: BMC Public Health. 2026 Jul 25;26:3020. doi: 10.1186/s12889-026-28725-y (PMC13685081; doi:10.1186/s12889-026-28725-y)
Supplement: Supplementary file 1 [file 12889_2026_28725_MOESM1_ESM.docx]

**Questionnaire**

| **SN** | **QUESTIONS** | **RESPONSE** | **INDICATE RESPONSE** |
| --- | --- | --- | --- |
| **Demographics** | | | |
| 1 | What is your gender? | a. Male b. Female |  |
| 2 | How old are you? |  |  |
| 3 | What is you highest level of education? | a. No formal education b. Basic c. JHS d. SHS e. Vocational training f. Tertiary |  |
| 4 | What certificate have you attained for practice? | a. Certificate b. Diploma c. First-Degree d. Post- Graduate |  |
| 5 | What type of facility do you work at? | a. Chemical shop b. Pharmacy store c. Health facility d. Herbal shop e. Other (specify) |  |
| 6 | What is your current job role? | a. Pharmacist b. Pharmacy technician c. Pharmacy assistant d. Medical counter assistant e. Other (specify) |  |
| 7 | How many years of work experience do you have? |  |  |
| 8 | Have you ever worked in a health facility as a dispenser? | a. No b. Yes |  |
| 9 | If **yes,** in **008,** for how long did you work in the facility? |  |  |

| **SN** | **QUESTIONS** | **RESPONSE** | **INDICATE RESPONSE** |
| --- | --- | --- | --- |
| **Antibiotics dispensing patterns in community drug outlets** | | | |
| 10 | How often do you dispense antibiotics? | a. Daily b. Weekly c. Monthly |  |
| 11 | Based on your answer in **010,** averagely how many times do you dispense antibiotics? | a. Once a. Twice b. Thrice c. Four times d. More than four times |  |
| 12 | Do you dispense antibiotics without prescription? | a. No b. Yes |  |
| 13 | How often do you experience a patient who asks for an antibiotic without a prescription? | a. Daily b. Weekly c. Monthly d. Once in a while e. Never |  |
| 14 | What will you do if patient asks for a specific antibiotic without prescription? | a. Ask for prescription b. Dispense without prescription c. Ask for the condition the person is buying the drug for d. Other (specify) |  |
| 15 | Which of the following antibiotics do you dispense often?  ***Select more than* *1*** | a. Amoxicillin + clavulanic acid b. Ampicillin c. Amoxicillin d. Cefixime e. Metronidazole f. Gentamicin g. Erythromycin h. Flucloxacillin i. Doxycycline j. Tetracycline k. Ceftriaxone l. Ciprofloxacin m. Ofloxacin n. Azithromycin o. Roxithromycin p. Cotrimoxazole q. Other (specify) |  |
| 16 | For which of the following diseases do you dispense antibiotics without prescription? | a. Genital and urinary b. Respiratory c. Gastrointestinal d. Dental e. Skin   Others (specify) |  |

| **SN** | **QUESTIONS** | **RESPONSE** | **INDICATE RESPONSE** |
| --- | --- | --- | --- |
| **Antimicrobial stewardship practices on antibiotic dispensing** | | | |
| 17 | Would you prescribe another antibiotic to a patient if the intended drug will not be effective for their condition? | a. No b. Yes |  |
| 18 | What will you do if the requested brand of antibiotic is not available in your pharmacy? | a. Replace with another brand b. Refer to another pharmacy c. Other (specify) |  |
| 19 | What advice do you give to patients about using antibiotics? | a. Complete full course b. Adhere to dosage c. Other (specify) |  |
| 20 | Do you request for feedback after dispensing antibiotics? | a. No b. Yes |  |
| 21 | What will you do if the patient has insufficient money to purchase an antibiotic? | a. Replace with a cheaper brand b. Reduce the quantity of antibiotics c. Other (specify) |  |
| 22 | Which of the following resources do you consult before dispensing antibiotics? | a. Clinical guidelines b. Pharmacy protocols c. Consultation with other healthcare professionals d. Patient's antibiotic use history c. Other (specify)… |  |
| 23 | Do you provide counselling to a patient about the appropriate use of antibiotics when necessary? | a. No b. Yes |  |
| 24 | For antibiotics that are used for lengthy periods, do you counsel the patient about their appropriate use? | a. No b. Yes |  |
| 25 | How do you handle situations where patients insist on antibiotics when not medically necessary? | a. Dispense anyway b. Not dispense c. Ask the patient to consult a physician d. Dispense based on my knowledge e. Dispense based on my experience f. Other (specify)… |  |

| **SN** | **QUESTIONS** | **RESPONSE** | **INDICATE RESPONSE)** |
| --- | --- | --- | --- |
| **Factors influencing antibiotic dispensing patterns** | | | |
| 26 | How do patients convince you to dispense antibiotics? | a. Belief in antibiotic efficacy b. Previous use c. Urgency and convenience d. Peer influence e. Other (specify) |  |
| 27 | Do you assess a patient’s medical history in situations where a patient is purchasing antibiotics without prescription? | a. No b. Yes |  |
| 28 | If **yes**, what are the records you assess? | a. Any medical condition b. Previous medication history c. Other (specify) |  |
| 29 | Which of the following influences you to dispense antibiotics? | a. Socioeconomic status of patient b. Education level of patient c. Pressure from other healthcare professionals d. Fear of negative patient feedback e. Patient's symptoms f. Patient's medical history g. Patient's ability to pay h. Profit i. Other (specify) |  |
| 30 | Do you feel pressure from patients to prescribe antibiotics? | a. No b. Yes |  |
